# Annotations

**Published:** 1905-04-08

**Authors:** 


### ANNOTATIONS.

### Sewage Pollution of Watercress.

At the meeting of the London County Council on Tuesday a report was presented which cannot fail to seriously alarm the consumers of watercress in the metropolis. The medical officers of the Council have submitted to the Public Health Committee the results of an exhaustive inquiry on the condition of watercress-beds in the County of London, and of the sources outside the county from which, within a radius of 50 miles, watercress is supplied to London. The need for the investigation and the importance of it, are emphasised by the fact that 1,500 tons of watercress are partaken of annually in the capital. The number of beds visited was 120, varying in size from under a quarter of an acre to nearly 40 acres. The report states that the conditions in which the beds are maintained differ considerably. In some cases special precautions are taken to prevent the danger of contamination, in others the beds are situated in positions which render them unlikely to be exposed to pollution. But in many it appears that surface drainage from grazing land is allowed to enter the beds, that offensive trades are carried on quite near to them, and an instance is cited of a case in which 25 or 30 houses without sewer accommodation are in close proximity to the bed. It would be a pity if, in consequence of this disturbing report, the demand for watercress entirely ceased. Grown under proper conditions it is a wholesome as well as a pleasant article of food. Nor is it fair that growers whose beds are pronounced free from contamination should suffer for the offences of those who sell plants which may have been impregnated with sewage. We therefore hope that the Local Government Board, to whom particulars of the investigations are to be transmitted, will promptly take steps to close the beds that constitute a peril to the health of the community. So long as they are allowed to continue sources of supply, watercress is likely to be severely let alone by all who do not wish to run unnecessary risks of contracting typhoid fever.

### Health Teaching in Elementary Schools.

The movement for the promotion of the teaching of hygiene and temperance in elementary schools has been visibly quickened by the recent conference of members of the medical profession, and by the presentation to the Board of Education of a memorial signed by nearly 15,000 medical men who asked for the intervention of the Board. The deputations who represented the latter were told that action was at present impracticable because the teachers in elementary schools are not as yet qualified to give instruction in hygiene. In these circumstances it is rightly urged that the training of teachers and the provision of suitable books are the first thing to be considered. It is certain that official excuses cannot be allowed to do more than temporarily block the way. The school curriculum is crowded already, but the health of the people is of the first importance, and in the face of the decline in the physical condition of the poorer classes as revealed in the report of the Inter-Departmental Committee on Physical Deterioration, it is impossible to deny that the matter is not pressing. It is hopeless, perhaps, to try to get at the insanitary adults who have become acclimatised to the surroundings which make for the spread of disease. Bad food, dirt, absence of ventilation, have been so long their daily portion that they have no desire for any improvement, especially if they have acquired the habits of intemperance which are fatal to aspirations after a higher standard of existence. But the children are accessible, and though there are in the country some schools in which a measure of instruction in hygiene is given, nothing short of health teaching as an essential portion of the system of education can render it possible to make the rising generation realise the vital importance of wholesome food, cleanliness, and fresh air. With regard to the teaching of temperance, it must not be forgotten that a good deal is done in this direction by the agency of Bands of Hope. But systematic instruction by qualified persons in hygiene would, we believe, tend to transform the conditions of the home life; it might reasonably be expected that in this way it would diminish temptations to intemperance as well as dissipate erroneous conclusions respecting the use of stimulants.

### Some Carnegie Trust Figures.

The figures which appear in the report of the Carnegie Trust for the Universities of Scotland for the year 1904 are calculated to excite envy in the breasts of educationalists whose schemes are hampered by the "eternal want of pence." It would seem that the trustees during the year had for distribution as grants to the universities and for the endowment of research no less a sum than £59,201. In addition, the income of the Trust included £50,000 to be utilised in the payment of the class fees of students who applied to the Trust and satisfied the necessary conditions. As a matter of fact some £46,000 was distributed for this latter purpose. The figures show that out of every hundred students 72 at Aberdeen received fees from the Trust, 70 at St. Andrews, 50 at Glasgow, and 39 at Edinburgh. This system of wholesale endowment of individual students has not yet been in operation sufficiently long to permit an estimate of its effects on education to be framed, but it is so far satisfactory to note that during the year six graduates who had in former years been beneficiaries under the Trust have refunded the class fees paid on their behalf. Mr. Carnegie, in his original scheme, expressed a confident opinion that the independent spirit of his countrymen would find expression in this fashion, and the justification of his confidence will doubtless be grateful to him. The contributions to the general funds of the Universities and to the encouragement of research must be wholly beneficial, and we can only wish that other universities enjoyed similar good fortune. The amount under the first of these heads in 1904 was over £38,000, and under the latter the sum of nearly £5,000 was distributed.

